# Relevance of level IIb neck dissection in oral squamous cell carcinoma

**DOI:** 10.4317/medoral.20491

**Published:** 2015-06-27

**Authors:** Juan-Carlos de Vicente, Tania Rodríguez-Santamarta, Ignacio Peña, Lucas Villalaín, Álvaro Fernández-Valle, Manuel González-García

**Affiliations:** 1 PhD. MD. Department of Oral and Maxillofacial Surgery, Hospital Universitario Central de Asturias, Oviedo, Spain; 2 PhD Instituto Universitario de Oncología del Principado de Asturias, Spain

## Abstract

**Background:**

The purpose of this study was to determine the prevalence of level IIb metastasis in patients with oral squamous cell carcinomas (OSCCs).

**Material and Methods:**

A prospective analysis of 56 patients with OSCC who underwent surgical treatment of the primary lesion with simultaneous neck dissection was performed. During neck dissection, level IIb lymph nodes were separately removed and processed. Neck dissection was bilateral in 26 patients (46%) and unilateral in 30 patients (54%).

**Results:**

The mean number of nodes found in the level IIb specimens was 4.7 (range: 0-8 nodes). The prevalence of metastasis at level IIb was 0% in pN0 necks and 3.4% in pN+ necks, with an overall prevalence of 1.8%. A significant association between metastasis to level IIb and type of neck dissection was observed. There were no isolated metastases to level IIb without the involvement of other nodes in the remaining neck specimen. Four regional recurrences were observed during follow-up.

**Conclusions:**

Based on our findings, we suggest that dissection of the level IIb region in patients with OSCC may be required only in patients with multilevel neck metastasis or if level IIa metastasis is found intraoperatively.

**Key words:** Oral squamous cell carcinoma, neck dissection, level IIb, metastasis, spinal accessory nerve.

## Introduction

**Figure 1:**
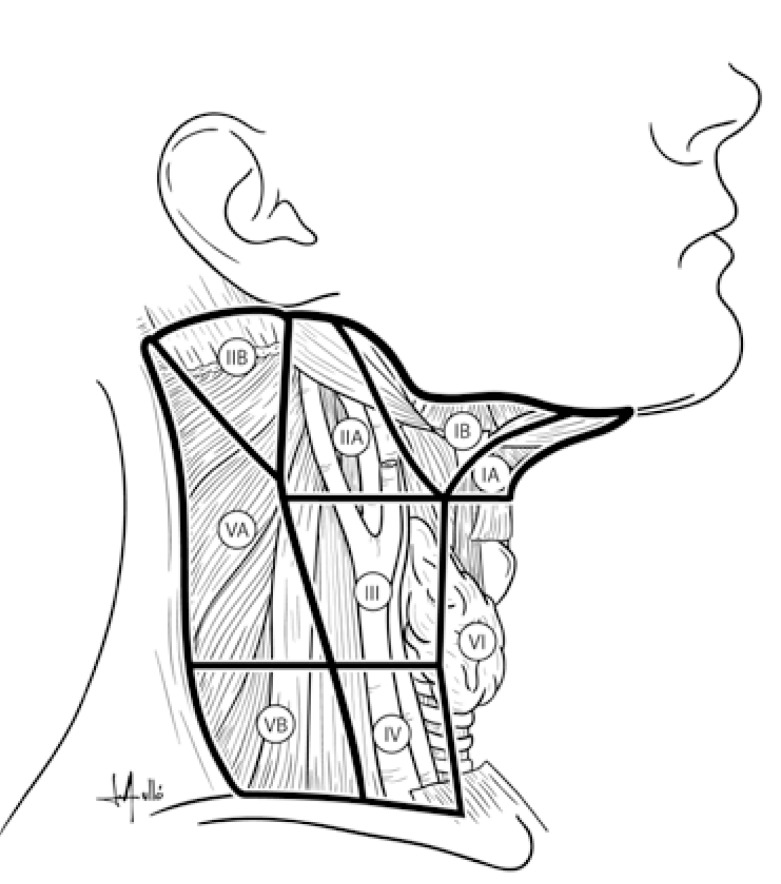
Leveling system of cervical lymph nodes: Level I contains the submental and submandibular nodes; it is bounded by the posterior belly of the digastric muscle and the hyoid bone inferiorly and by the body of the mandible superiorly. Level II contains the upper jugular lymph nodes and extends from the skull base superiorly to the hyoid bone (clinical landmark) or the bifurcation of the carotid artery. Level III contains the mid-jugular lymph nodes and it extends from the inferior border of level II up to the lower border of the cricoid cartilage (clinical landmark) or the omohyoid inferiorly. Level IV contains the lower jugular lymph nodes; it extends from the inferior border of level III up to the clavicle inferiorly. The anterior border of levels II, III, and IV is the lateral limit of the sternohyoid muscle, and the posterior limit of these levels is the posterior border of the sternocleidomastoid muscle. Level V contains the lymph nodes in the posterior triangle. It is bounded by the anterior border of the trapezius posteriorly, the posterior border of the sternocleidomastoid muscle anteriorly, and the clavicle inferiorly. Level VI contains the anterior lymph nodes, extending from the hyoid bone superiorly up to the suprasternal notch inferiorly. Its lateral border is formed by the medial border of the carotid sheath.

Oral squamous cell carcinoma (OSCC) is a life-threatening disease which represents 32-40% of all the head and neck cancers (HNC) (1). The biological aggressiveness of oral cancer is reflected by its ability to lead to neck lymph nodal metastasis (N+). The work of Weiss *et al*. (2) established a threshold of a 20% possibility of cervical metastasis as the indication for elective treatment of the neck in squamous HNC. Although conflicting rationales exist regarding the most appropriate therapeutic management of a clinically N0 OSCC, and based on the observation that lymph node metastasis from carcinomas of the oral cavity tend to exhibit a typical pattern of spread, most frequently to levels I, II, and III (3) (Fig. 1), a supraomohioid neck dissection (SOHND) is usually proposed for the treatment of OSCC in N0 cases. The surgical therapy of patients with oral carcinomas and clinical N+ neck is less controversial than the treatment of the clinical N0 neck. Currently, many surgeons worldwide still perform standard radical neck dissection (RND) or modified radical neck dissection (MRND) in cases of clinical N+ neck (4). Unlike RND, SOHND preserves important anatomic structures, such as the spinal accessory nerve (SAN), while also maintaining oncologic safety. One of the more technically difficult aspects of ND is the dissection of the upper inner jugular vein and the SAN lymph nodes, in the posterior region of level II. This area is known as level IIb (5), but it also has been called supraretrospinal recess (6) and submuscular recess (SMR) (7). Level IIb comprises node-bearing tissue that lies superficial to the fascia on the splenius capitis and levator scapulae muscles, and is bordered anteroinferiorly by the SAN, superolaterally by the inferior border of the posterior belly of the digastric muscle, superiorly by the skull base, and posterolaterally by the sternocleidomastoid muscle. Complications that may present during and after level IIb dissection include SAN dysfunction, which results in the limitation of shoulder movements (8) and thus has a negative impact on quality of life. To overcome this complication, avoiding the dissection of level IIb has been proposed. There still remains a debate about the frequency of metastasis to level IIb, and despite various published works, the question of whether or not to dissect level IIb remains to be clarified. Thus, the aim of this study was to determine the frequency of level IIb metastasis in OSCC by means of a prospective study in order to assess whether level IIb dissection should be performed or may be avoided in the treatment of this disease.

## Patient and Methods

This prospective study involved 56 previously untreated consecutive patients with OSCC who were treated at the Department of Maxillofacial Surgery of Hospital Universitario Central de Asturias (Oviedo, Spain), enrolled between January 2010 and November 2011. In order to be included in the study, eligible patients had to have undergone surgical resection of a primary OSCC, in conjunction with a unilateral or bilateral, elective or therapeutic, neck dissection that included sub level IIb. This 56 patients represent the 77% of the patients treated during this period of time. Exclusion criteria were synchronous head and neck cancers, a history of surgery or radiotherapy of the head and neck, a history of head and neck cancer, and primary treatment with radiotherapy. Patient and tumor characteristics are provided in  Table 1. Tobacco and alcohol consumption history was determined from medical records. Alcohol consumption was described in terms of beer, wine or hard liquor. A drinker was defined as a person who self-reported consuming four or more drinks of any alcoholic beverage type per day for at least 10 years. A smoker was a patient who self reported consuming five or more cigarettes per day for at least five years. All diagnoses were confirmed histopathologically before treatment, and the disease was staged after the surgical resection of the tumor according to the TNM system of the American Joint Committee on Cancer. After tumor resection, a reconstruction was performed in 28 cases (50%), an anterolateral thigh flap was used in ten cases, a fibular flap in eight cases, a forearm free flap in seven cases, a scapular flap in one case, a sural flap in one case, and two flaps (forearm plus fibulas) in one case. The extent of the ND was modulated according to the clinical neck status. The fibrofatty tissue of level IIb was dissected and removed before completing the remainder of the ND, and then labeled and processed in a separate box for histopathologic analysis. The ND was subsequently completed and the remaining neck specimen was divided and labeled as I, IIa, III, IV, and V level by the surgeon before being submitted for pathological analysis. Postoperative radiotherapy was applied to 24 (43%) patients. These patients either had pT4 tumors, positive lymph nodes, or margins of less than 4 mm. Radiotherapy was initiated within 4 to 8 weeks after surgery. A total dose of 60-70 Gys was delivered in two Gys per-fraction. The follow-up period ranged from 7 to 38 months. The regional recurrence rate with control of the primary site was also determined.

**Table 1:**
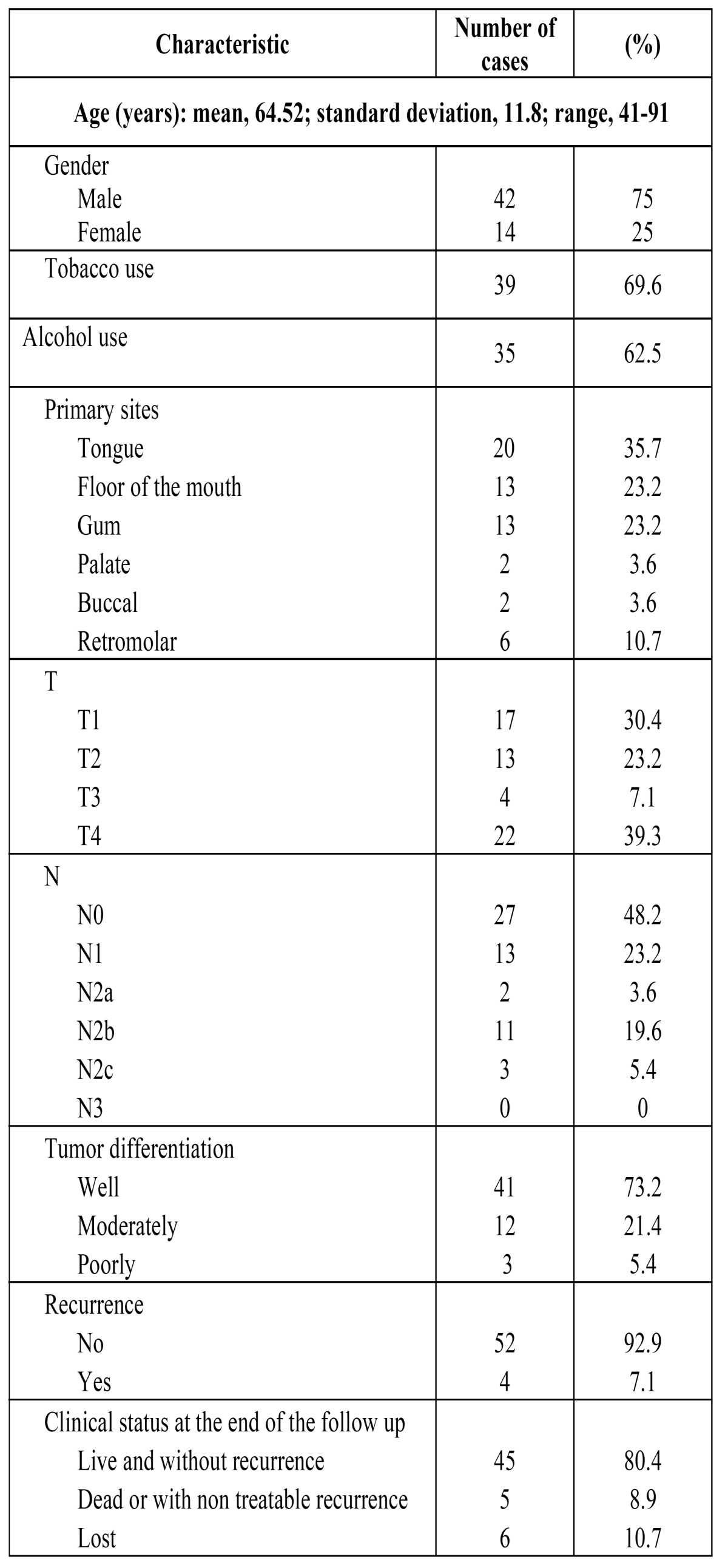
Clinical features in 56 patients with oral squamous cell carcinoma.

This study was performed with the permission of the Ethical Committee of the Hospital Universitario Central de Asturias and an informed consent was obtained from all patients. Statistical analysis was performed using the commercially available computer software package PASW Statistics 18 (2009 SPSS Inc.). Categorical data were compared using the Pearson χ2 test or Fisher’s exact test when at least one cell had an expected frequency of less than five. *P* values ≤0.05 were considered statistically significant.

## Results

Overall, 56 patients were included in the study and 82 neck dissections were performed in total. The types of neck dissections and staging according to the AJCC are shown in  Table 2. The number of lymph nodes removed during ND ranged between 9 and 41, with an average of 18.4 lymph nodes. The mean number of nodes found in the level IIb specimens was 4.7 (range 0-8 nodes). The neck node specimens of 29 (51.8%) patients were pathologically N+; among them, 18 patients had a single positive node, six patients had two positive nodes, two patients showed three metastatic neck nodes, and three patients had four positive nodes each. Thirty-five neck lymph nodes were harvested from ipsilateral level IIa in all 56 patients. Only one of these patients had metastases in level IIb, specifically, 3 lymph node metastasis from 5 isolated lymph nodes in this level, representing 1 out of 56 patients (1.8%) and 1 out of 82 neck dissection specimens (1.2%), combining pathologically N0 necks and clinically node-positive necks. The aforementioned IIb positive patient had an associated synchronous 4-neck node metastasis in ipsilateral node levels IIa and III. She was an 85-year-old female who presented with a well-differentiated pT1pN2b right-side tongue cancer ( Table 3). In no case were there metastases at level IIb without the involvement of other lymphatic chains. Regarding the tumor site, only one tumor located in the tongue showed metastasis in the level IIb lymph nodes, while the remaining tongue cancers and oral cavity locations did not show association with level IIb involvement.

**Table 2:**
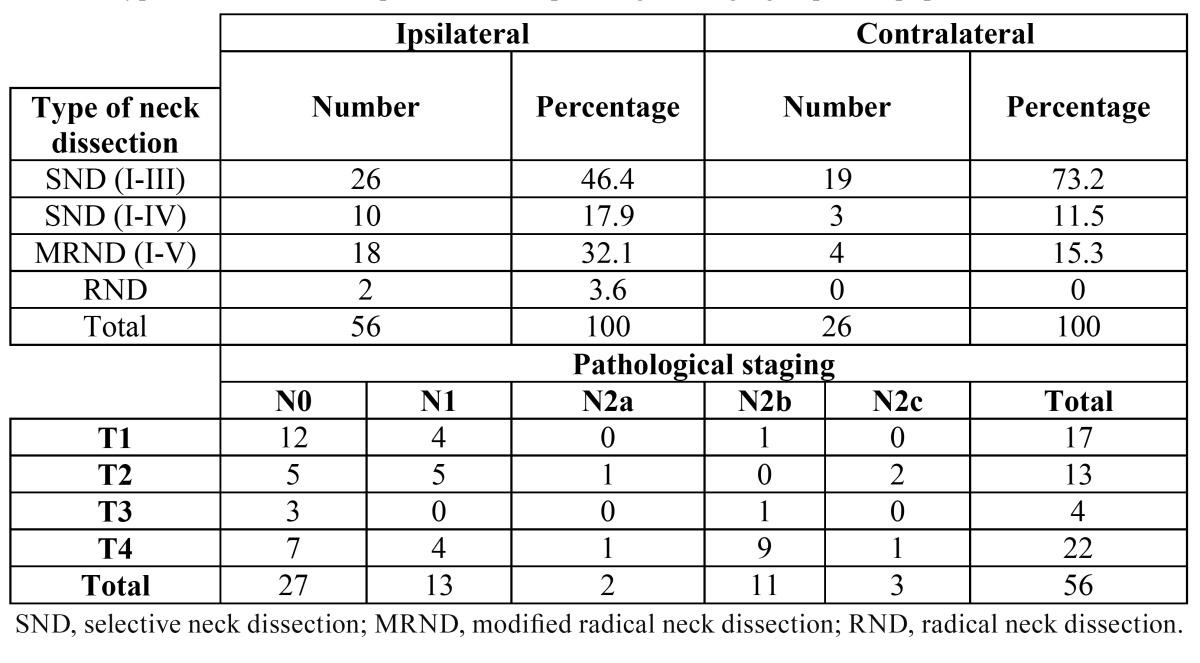
Type of neck dissection performed and pathological staging of patient population.

**Table 3:**
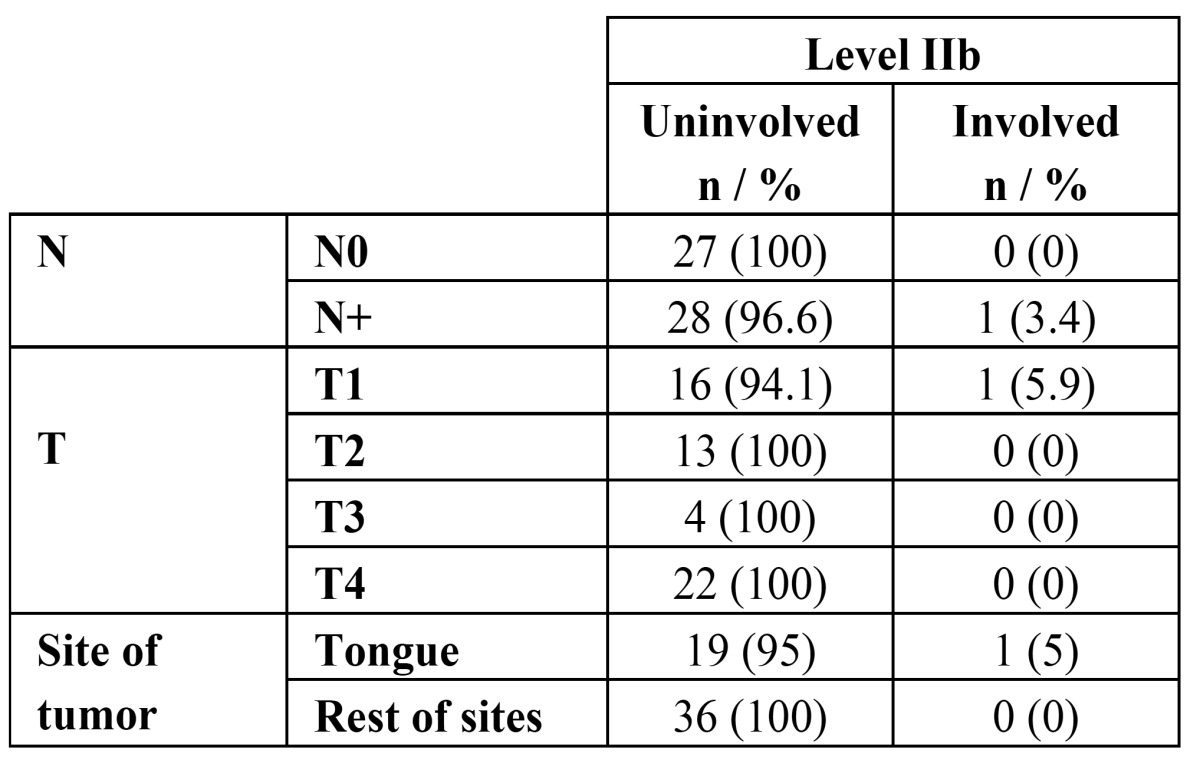
Level IIb metastasis ratios by neck node status, tumor stage and tumor site.

Several variables were included in the analysis of potential risk factors for nodal metastasis at level IIb. There was no association between any neck levels with pN+ and the occurrence of pN+ at level IIb. Furthermore, it was not possible to demonstrate the influence of the number of histopathologically positive neck levels over the presence of metastases at level IIb through histopathologic evaluation ( Table 4). A total of four regional recurrences (7.1%) were documented during patient follow-up. No statistically significant association between the presence of nodal metastases at level IIb and advanced pathological N stage was observed (*p*=0.5), but in no instance were there positive nodes at level IIb without metastatic disease at one other nodal level at least. Only a significant relationship between metastasis to level IIb and type of neck dissection (*p*=0.03) was observed ( Table 4). Because of the small number of ND with positive nodal disease at level IIb, no multivariate analysis was performed.

**Table 4:**
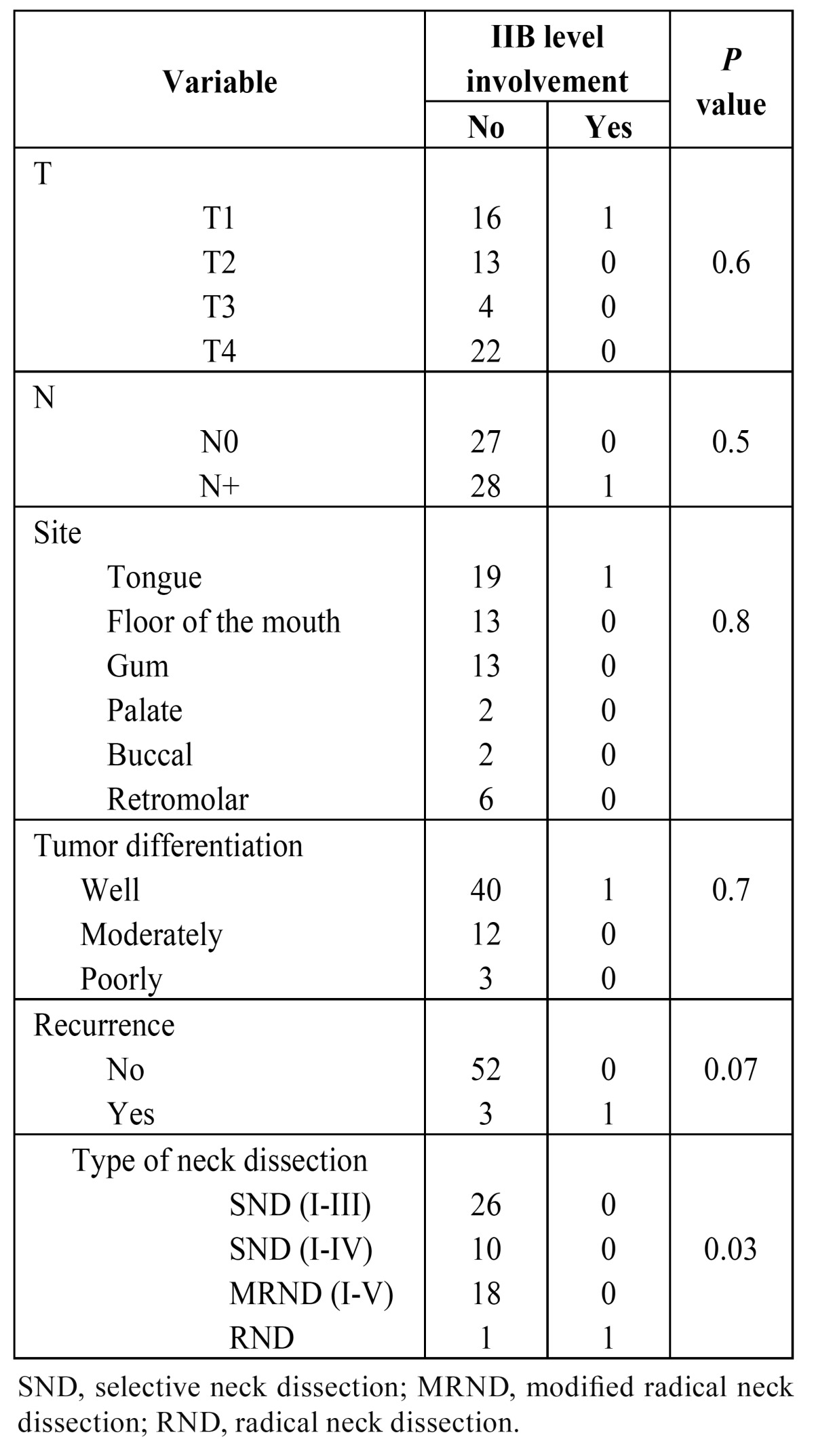
Analysis by χ2 test or Fisher exact test on the association between six variables on the prevalence of level IIb metastasis.

## Discussion

The lymphatic spread of oral cancer cells to the neck usually follows a well-known pattern. Ninety-one percent of neck node metastases from oral cancers are concentrated in levels I to III, and if level IV is added to the I-III levels in a selective neck dissection, 96% of all nodal metastases from oral cancers would be covered in this dissection. The overall incidence of metastasis from OSCC to level V is as low as 3.3% (9). Level IIb lymph node dissection has been performed as part of elective neck dissection since Schuller *et al*. (10) observed the high nodal metastasis rate near the SAN. Nevertheless, dissection of level IIb in OSCC is controversial nowadays. It is well-known that the oral cavity does not drain directly into level IIb (8,11,12). The controversy arises from the low reported risk of level IIb nodal metastasis and potential shoulder disability. In selective neck dissections, such as SOHND, shoulder function impairment has been observed in 21% to 60% of cases instead of the preservation of the SAN (13). Erisen *et al*. (14) observed that in all types of neck dissections all patients exhibited electromyographic evidence of denervation when compared with a control group. At about the third postoperative month, the nerve function returns (15), but reparation of axonal damage can take from 12 to 18 months. Kierner *et al*. (16) have reported that the SAN passed dorsally to the internal jugular vein in 44% of cases and ventrally in 56% of cases. Lee *et al*. (17) showed that the SAN courses dorsally to the vein in 57.4% of cases, ventrally in 39.8% of cases, and through the internal jugular vein in 2.8% of cases. The number of lymph nodes at level IIb varies from patient to patient and throughout the course of the nerve. Lee *et al*. (17) observed that the mean number of lymph nodes at level IIb was 6.5 when the SAN crossed ventrally and 6.8 in those cases in which the SAN crossed the internal jugular vein dorsally.

Recent studies that look for the frequency of metastasis at level IIb in N0 OSCC found figures that ranged from 0% to 22%, with 95% confidence intervals (0% to 44.4%) (18). Chone *et al*. (19) performed a retrospective analysis of 51 patients with different carcinomas of the upper aerodigestive tract and found metastatic lymph nodes in the sub muscular recess in 4 of 62 neck dissections. Three of these patients were N2 and one patient suffered from a T2N0 carcinoma of the floor of the mouth. However, it is well-known that lymphatic metastatic spread patterns from different primary sites can vary greatly (12), and it is hard to estimate the prevalence of metastasis in a heterogeneous group of patients with different primary sites instead of only surgically treated cases as initial therapy. Our study group is homogeneous in terms of location. All tumors are located in the oral cavity, and they tend to exhibit a typical pattern of spread, mainly to levels I, II, and III (3). Talmi *et al*. (20) reported an incidence of 6% of occult metastasis at level IIb in patients with OSCC who underwent elective ND. Lim *et al*. (21) reported that level IIb nodal metastasis was present in four (5.4%) of 74 cases of cN0 OSCC in which an SOHND was performed. Elsheikh *et al*. (22) found a prevalence of 10% at level IIb in a molecular study on 48 patients with OSCC and N0 neck who underwent a SOHND. Villaret *et al*. (23) stated that the oral cavity has the highest overall prevalence of level IIb metastasis (10%) among head and neck squamous cell carcinomas, but these metastases are only found in 2% of cN0 cases. In a recent study, Lea *et al*. (18) observed that the frequency of level IIb nodal metastasis ranges in the literature from 0% to 10.4%, with 95% confidence intervals (CI) of 0% to 44.4% (19). Based on a meta-analysis, the authors concluded that the frequency of nodal metastasis to level IIb in previously untreated OSCC is 6.0% (95% CI: 3.5-8.6).

The primary limitation of our study, and all others published to date on this topic, is the sample size, as well as the limited follow-up. However, despite the sample size, we have obtained some relevant results. The incidence rate of neck lymph node metastasis in patients who underwent an elective SOHND for OSCC was 51.8%. Lymph node metastases were detected at level IIb in one of 56 patients with OSCC, which means a prevalence of 1.8%, lower than in other studies, and the percentage of metastasis at level IIb in N0 cases was 0%. In our series, isolated metastases were not found at level IIb. When compared with the current literature, this is a low prevalence, and in addition we did not found any association between clinical variables and metastases at level IIb. It would appear that T stage and histological grade of differentiation, as well as tumor location within the oral cavity are not reliable indicators of metastasis at level IIb. One reason may be that more than 50% of patients had T1 and T2 carcinomas, and 94% of them were well or moderately differentiated. Therefore, a limitation of this study could be the statistical power due to the relatively small number of patients. When an SOHND was performed in cN0 oral cancer patients who had been surgically treated as initial therapy, occult nodal metastasis to level IIb did not occur on either the ipsilateral or contralateral neck. However, when ipsilateral node metastasis was present, specifically at level II, the cervical metastatic rate to ipsilateral level IIb lymph nodes was 3.4%. In this study, the patient with metastasis at level IIb also had metastasis at levels IIa and III.

We found four regional recurrences during follow-up, one of them in the patient with a tongue carcinoma and metastasis to level IIb. Occult metastases are tumoral lymph node deposits undetected by clinical or radiographic examination, and they can be further subdivided into three types: macro metastasis (greater than 2-3 mm in largest dimension), micro metastasis (smaller than 2-3 mm in largest dimension), and isolated tumor cells within the lymph nodes (24). In HNC, recurrence rate is approximately 10% in patients who had histopathologically negative neck dissection specimens, suggesting that metastases were present but not detected in the resected specimens (25). This result was comparable to those in Ferlito *et al*. (26), in which a range of 8% to 20% of neck nodal metastases in patients with HNC were not identified by routine histologic examination (27) and were consequently underestimated. Elsheikh *et al*. (22), using molecular analysis, found an incidence of occult metastasis to level IIb in 5 of 23 (22%) patients with primary tumors located in the tongue. Thus, they defend that level IIb should be included in the neck dissection in cases of tongue cancer. In our current study, the only level IIb metastasis case found was observed in a patient afflicted with tongue cancer. Due to the fact that our sample included 20 tongue carcinomas, the prevalence of metastasis in this subgroup of oral cavity carcinomas is 5%, lower than the 20% prevalence required to recommend elective treatment. Level IIb metastases are rarely found in isolation; instead, IIb metastases are most often found combined with occult or gross pathologic nodes at other levels. Maher and Hoffman (28) found cervical sublevel IIb lymph node metastases in 4 (5.6%) of 71 patients with primary OSCC. Three of these IIb metastases were found in patients with tongue carcinomas, and the other case in a patient afflicted from a cancer located in the retromolar trigone. Pantvaidya *et al*. (9) described similar findings: tongue cancers (5%) and cancers of the retromolar trigone (6.2%) had the highest incidence of metastasis to level IIb. They also found that metastases at level IIb were associated with metastases at level IIa in 68.1% of dissections; conversely, only 11.3% of all level IIa metastases had positive nodes at level IIb.

Based on our findings, and considering the low incidence of level IIb nodal metastases in OSCC, we suggest that it may be unnecessary to resect level IIb lymph nodes for most people with OSCC when performing an elective neck dissection in cN0 cases and, consequently, postoperative shoulder disability may be avoided. Thus, dissection of level IIb in patients with OSCC may be required only in patients with multilevel neck metastasis or if level IIa metastasis is found intraoperatively. However, because only one patient had sublevel IIb metastasis in this study, our results would require additional validation, and its significance should be interpreted cautiously. The high metastatic rate of patients with clinically positive neck nodes, mainly in patients staged N2b or greater (29) confirms the recommendation given by some authors (19) to always dissect level IIb in these cases, considering that the optimal moment to deal with these nodes is at the time of initial treatment. Elsheikh *et al*. (22) also advocate including level IIb in elective dissection whenever the tongue is the primary tumor site.
